# Supplementary material for: Nicotine Exposure during Adolescence Leads to Changes of Synaptic Plasticity and Intrinsic Excitability of Mice Insular Pyramidal Cells at Later Life
Source: Int J Mol Sci. 2021 Dec 21;23(1):34. doi: 10.3390/ijms23010034 (PMC8744609; doi:10.3390/ijms23010034)
Supplement: Supplementary file 1 [file ijms-23-00034-s001.zip › ijms-1495270-supplementary.pdf]

Supplemental Material

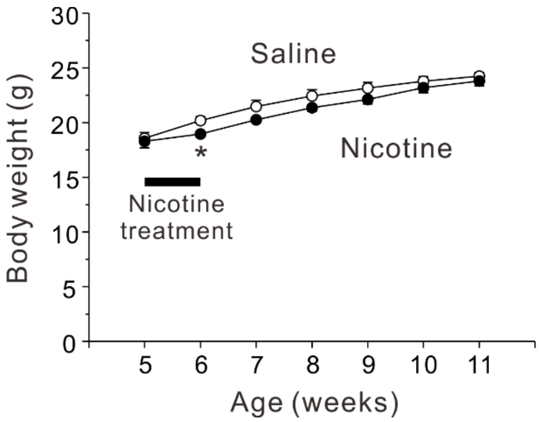

Supplementary Figure S1. Effects of adolescent nicotine exposure on body weight in mice. Summary data of body weight obtained from saline-treated mice (open circle, n = 6) and nicotine-treated mice (filled circle, n = 7). Mice just after one week nicotine exposure (6 weeks of age) had significantly less body weight than saline-treated mice (p = 0.036). Before and 1, 2, 3, 4 and 5 weeks after the nicotine treatment, there was no significant difference in body weight between two groups (5 weeks of age, p = 0.674; 7 weeks of age, p = 0.074; 8 weeks of age, p = 0.113; 9 weeks of age, p = 0.126; 10 weeks of age, p = 0.373; 11 weeks of age, p = 0.506). Two-way repeated measures ANOVA post-hoc LSD, *: $p < 0.05$.
